# Supplementary figures and images for: A Sensor and Machine Learning-Based Sensory Management Recommendation System for Children with Autism Spectrum Disorders
Source: Sensors (Basel). 2022 Aug 3;22(15):5803. doi: 10.3390/s22155803 (PMC9371185; doi:10.3390/s22155803)

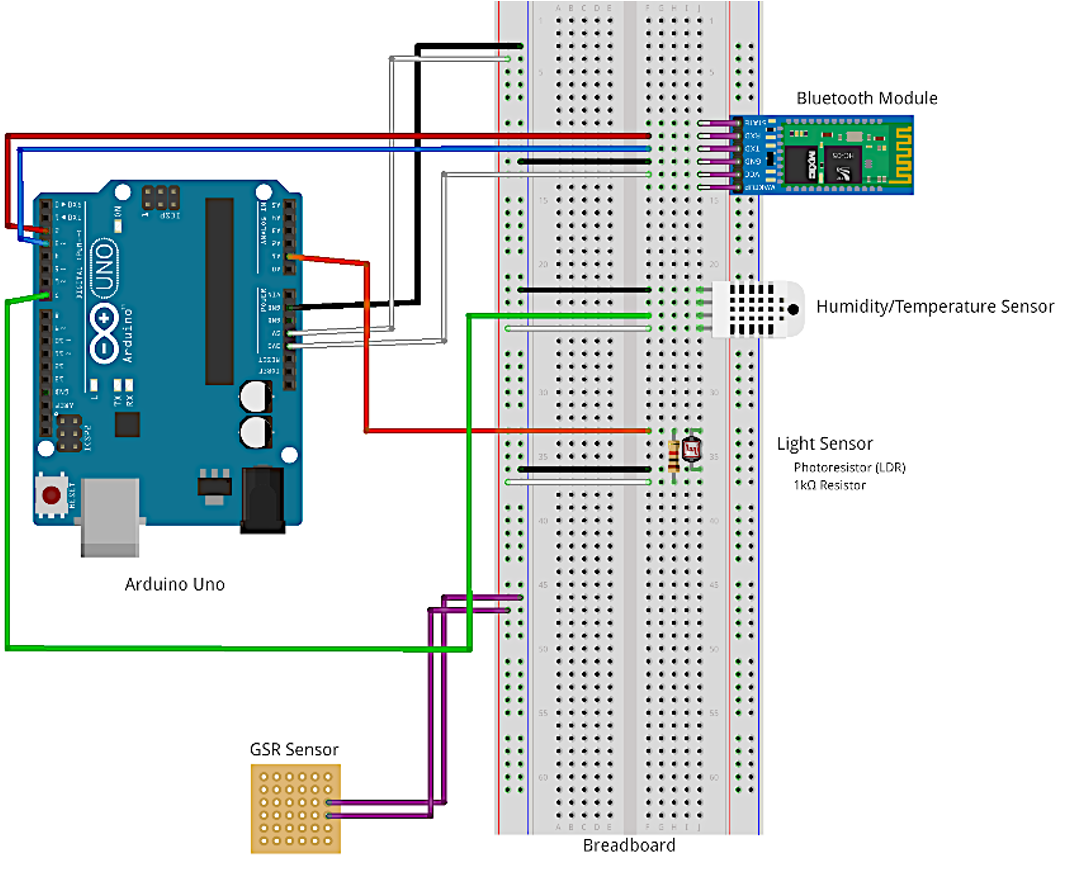

Supplement: Supplementary file 1 [file sensors-22-05803-s001.zip › FigureS1.png]

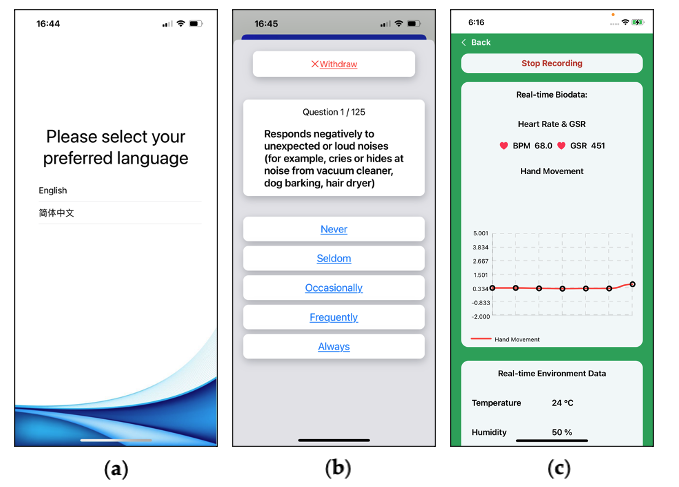

Supplement: Supplementary file 1 [file sensors-22-05803-s001.zip › FigureS2.png]
